# Qualitative analysis of shared decision-making for chemoprevention in the primary care setting: provider-related barriers

**DOI:** 10.1186/s12911-022-01954-y

**Published:** 2022-08-04

**Authors:** Tarsha Jones, Thomas Silverman, Ashlee Guzman, Julia E. McGuinness, Meghna S. Trivedi, Rita Kukafka, Katherine D. Crew

**Affiliations:** 1 Christine E. Lynn College of Nursing, Florida Atlantic University, 777 Glades Road, Boca Raton, FL 33431 USA; 2 Department of Medicine, Vagelos College of Physicians and Surgeons, Columbia University Irving Medical Center, New York, NY USA; 3 Department of Epidemiology, Mailman School of Public Health, Columbia University Irving Medical Center, New York, NY USA; 4 Herbert Irving Comprehensive Cancer Center, Columbia University Irving Medical Center, New York, NY USA; 5 Department of Biomedical Informatics, Vagelos College of Physicians and Surgeons, Columbia University Irving Medical Center, New York, NY USA; 6 Department of Sociomedical Sciences, Mailman School of Public Health, Columbia University Irving Medical Center, New York, NY USA

**Keywords:** Breast cancer prevention, Chemoprevention, Shared decision-making, Minority women

## Abstract

**Background:**

Chemoprevention with anti-estrogens, such as tamoxifen, raloxifene or aromatase inhibitors, have been shown to reduce breast cancer risk in randomized controlled trials; however, uptake among women at high-risk for developing breast cancer remains low. The aim of this study is to identify provider-related barriers to shared decision-making (SDM) for chemoprevention in the primary care setting.

**Methods:**

Primary care providers (PCPs) and high-risk women eligible for chemoprevention were enrolled in a pilot study and a randomized clinical trial of web-based decision support tools to increase chemoprevention uptake. PCPs included internists, family practitioners, and gynecologists, whereas patients were high-risk women, age 35–75 years, who had a 5-year invasive breast cancer risk ≥ 1.67%, according to the Gail model. Seven clinical encounters of high-risk women and their PCPs who were given access to these decision support tools were included in this study. Audio-recordings of the clinical encounters were transcribed verbatim and analyzed using grounded theory methodology.

**Results:**

Six primary care providers, of which four were males (mean age 36 [SD 6.5]) and two were females (mean age 39, [SD 11.5]) and seven racially/ethnically diverse high-risk female patients participated in this study. Qualitative analysis revealed three themes: (1) Competing demands during clinical encounters; (2) lack of knowledge among providers about chemoprevention; and (3) limited risk communication during clinical encounters.

**Conclusions:**

Critical barriers to SDM about chemoprevention were identified among PCPs. Providers need education and resources through decision support tools to engage in risk communication and SDM with their high-risk patients, and to gain confidence in prescribing chemoprevention in the primary care setting.

**Supplementary Information:**

The online version contains supplementary material available at 10.1186/s12911-022-01954-y.

## Introduction

Breast cancer is the most common cancer and the second leading cause of cancer-related death among women in the U.S, making it an important public health priority [1]. The U.S. Preventive Services Task Force (USPSTF) recommends that clinicians offer to prescribe risk-reducing chemoprevention medications, such as selective estrogen receptor modulators (SERMs; tamoxifen, raloxifene) or aromatase inhibitors (AIs; anastrozole, exemestane), to women who are at high-risk for developing invasive breast cancer [2]. Although breast cancer chemoprevention medications, can reduce breast cancer incidence by up to 40–65% among high-risk women, less than 5% of eligible women take chemoprevention or breast cancer risk-reducing pills [3–5]. Reasons for low uptake have been previously described [6]; however, patient preferences and provider recommendation are strong factors that influence chemoprevention decision-making [7].

Shared decision-making (SDM) is a collaborative process by which healthcare providers partner with patients to achieve informed health decisions [8, 9]. Through SDM, the clinician ensures that the patient understands the risk of a disease or condition that is to be prevented, while discussing benefits and harms of preventive interventions (risk communication) and values clarification [10, 11]. Ideally, SDM follows a process of patient empowerment and consumer choice and moves away from the paternalistic nature of provider-driven care towards patient-centered care [12]. However, numerous provider-related barriers to SDM have been documented, including insufficient time during clinic appointments and minimal SDM competencies [10, 13]. Many clinicians lack training in interviewing techniques and struggle with organizing scientific evidence about the harms and benefits of preventive services [14]. Studies have found that even providers who are well-intentioned may experience challenges with SDM for chemoprevention due to limited time during office visits, competing demands, and finding time for chronic disease prevention [8, 12]. In comparison to these previous studies, the added value of our study is that we present data from real clinical encounters of providers and their high-risk patients in the primary care setting, most of whom are racial/ethnic minority women. The research gap that we hope to fill is a greater understanding of factors contributing to the low uptake of chemoprevention among high-risk women, particularly in the primary care setting where prevention of health issues should be a priority. The population who will benefit most from our study are women who are at increased risk for developing breast cancer, particularly minority women who suffer higher mortality rates from breast cancer.

In order to address barriers to SDM in relation to breast cancer chemoprevention in the primary care setting, our team developed two web-based decision support tools: *RealRisks* for patients and *BNAV* (Breast cancer risk NAVigation) for primary care providers (PCPs), which were both used in this current study. Results from an initial single-arm pilot study with 50 high-risk women showed that exposure to *RealRisks* led to improved accuracy of breast cancer risk perceptions and increased chemoprevention knowledge [15]. In this current study, which is a part of a larger randomized controlled trial (RCT) [3, 16],we aim to identify provider-related barriers to SDM for chemoprevention within clinical encounters occurring in the primary care setting.

## Methods

### Study design

Our study design is a qualitative study with clinical encounters of providers and their high-risk patients. The clinical encounter data was captured from providers enrolled in a pilot study that was conducted prior to implementing the RCT [15], the same providers were also enrolled in our main randomized controlled trial that was recently published [16]. Participants were eligible to participate in the RCT if they were high-risk women, with a 5-year invasive breast cancer risk ≥ 1.67% according to the Gail model or personal history of lobular carcinoma in situ (LCIS), and between the ages of 35–75 years, no prior SERM or AI use, English or Spanish speaker, and had a healthcare provider at Columbia University Irving Medical Center (CUIMC). These women were identified during screening mammography at CUIMC. Primary care providers (PCPs) enrolled in the RCT were internists, family practitioners, and gynecologists, nurse practitioners, and medical residents who saw high-risk women in their primary care clinics at CUIMC.

For this qualitative study, a subset of patients and providers enrolled in the pilot study and RCT were consented to have their routinely scheduled clinical encounters audio-recorded at CUIMC [3, 15]. All providers were given access to the *BNAV* tool and patients in the intervention arm were randomized to *RealRisks.* All audio-recordings of providers and their high-risk female patients were transcribed verbatim to capture the conversations occurring in the clinical encounters. Several of the encounters included Spanish-speaking patients and their interpreters. These encounters were transcribed in Spanish and then translated to English by bilingual members of the research team. The transcripts were then verified and de-identified by T.S. and T.J. Written informed consent was obtained from all enrolled patients and healthcare providers. These studies were approved by the institutional review board at CUIMC in New York, NY.

### Study setting

Patients in the intervention group of the RCT were randomized to *RealRisks*, a patient-centered decision aid (DA) that is available in English and Spanish. *RealRisks* includes interactive modules designed to improve a high-risk woman’s accuracy of her personalized breast cancer risk and increase her self-efficacy when engaging in dialogue about breast cancer risk and chemoprevention options with her healthcare provider. Through the *RealRisks* DA, patients inputted information on breast cancer risk factors to calculate their personalized 5-year invasive breast cancer risk according to the Gail model, learned about the risks and benefits of chemoprevention, and indicated their intention to take or not take chemoprevention and factors that influenced their decision-making. *RealRisks* then generated an action plan for patients summarizing their interactions with the DA. Healthcare providers had access to an intervention called *BNAV*. The *BNAV* tool is a repository of information and resources on breast cancer risk assessment and risks and benefits of chemoprevention, as well as a dashboard of personalized breast cancer risk reports for their enrolled patients [3, 15, 17]. *BNAV* includes educational modules on breast cancer risk, chemoprevention, screening, genetic testing, and risk communication. The patient and provider tools relate to each other as the patient inputs her information into *RealRisks* and the *BNAV* tool includes a summary of the patient’s personalized breast cancer risk profile and the patients’ personal preferences about chemoprevention, which are both made available to the provider through the *BNAV* tool. The efficacy of both tools is currently being tested in two randomized controlled trials [3, 16, 18].

### Data analyses

To generate a table of patients and provider characteristics, we conducted descriptive analyses to generate frequencies for categorical variables and means for continuous variables. For the qualitative analysis, seven audio-recorded clinical encounters between PCPs and their patients in the primary care setting were included. An investigator (TJ) trained in qualitative data analysis organized and coded the data using grounded theory methodology [19–21]. Coding with grounded theory involves an iterative process [19–21]. The first step was an initial open coding, where the researcher generated as many codes as possible, inductively [21]. In the second step, the investigator used memos and constant comparative method, an analytical process used in grounded theory for coding and category development. During the third step, advanced coding was used to identify central themes that answered the research question. NVIVO software (Version 10) was used to manage the data and to conduct the qualitative analyses. All co-authors were involved in selecting and approving the final excerpts of the clinical encounters that were included in the results section.

## Results

Table 1 summarizes the characteristics of six providers healthcare providers who participated in the clinical encounters. Four providers were males, mean age 36 years (standard deviation [SD], 6.5) and two providers were females, mean age 39 years, (standard deviation [SD], 11.5). Two providers were in residency and had not completed training as yet; one provider had 5–10 years’ experience; two providers had 16–20 years’ experience; and one provider had greater than 20 years’ experience. Table 2 provides a brief summary of the patients (N = 7) who participated in the clinical encounters with their providers. Most were Spanish-speaking Hispanic women (N = 5), with a mean age of 73 years (standard deviation [SD], 3.70). Most women had a high school education or less (N = 5).

**Table 1:** Baseline characteristics of internal medicine providers who were included in clinical encounters audio recordings with their patients (N = 6)

Characteristic	Female (N = 2)	Male (N = 4)
*Age, mean (SD)*	39 (11.5)	36 (6.5)
*Race/ethnicity*		
Non-Hispanic white	2	0
Non-Hispanic black	0	0
Hispanic	0	1
Asian	0	1
Other/unknown	0	2
*Education level*		
Medical degree	2	4
*Years of training*		
Have not yet completed training	1	1
< 5	0	0
5–10	0	1
11–15	0	0
16–20	1	1
> 20	0	1

**Table 2:** Baseline characteristics of patients who were included in clinical encounters audio recordings with their providers (N = 7)

Characteristic	English speaking (N = 2)	Spanish speaking (N = 5)
*Age, mean (SD)*	71 (2.83)	73 (3.70)
*Race/ethnicity*		
Non-Hispanic white	0	0
Non-Hispanic black	1	0
Hispanic	0	5
Other	1	0
*Education level*		
High school or less	0	5
Some college	1	0
Bachelors	1	0
Graduate school or higher	0	0

### Qualitative results

Based on our analysis and interpretation of the clinical encounter data, we identified three themes: (a) competing demands during clinical encounters; (b) lack of knowledge about chemoprevention; and (c) limited risk communication during clinical encounters.

#### Theme 1: competing demands during clinical encounters

In our study, we found that a key barrier to implementing SDM during the clinical encounter is that PCPs frequently addressed multiple health problems occurring with their patients and made many referrals for primary care health issues (Table 3); therefore, providers focused on establishing priorities with their patients due to competing demands. Clinical encounters appeared to be dominated by provider-driven conversations. For example, in one encounter the patient initiated a conversation about her breast cancer risk and the provider made the decision that he/she would only order a mammogram. The provider explained to the patient that with her history she does not need to take any more pills. The provider discourages the patient from taking medications by stating that she is “forgetful” and does not currently “take medication the way [she is] supposed to.” The provider states that blood pressure and obesity are the more pressing concerns. The conversation between the patient and the provider, as shown in Additional file 1: Box 1, appears to be provider-driven without engaging the patient in SDM. The patient may not have been aware that there is a decision to be made. The same clinical encounter is shown in Additional file 1: Box 2, and illustrates how the provider addresses primary care concerns and establishes priorities during a routine clinical visit.

**Table 3:** Types of decisions and referrals made during the Clinical Encounters

Taking a chemoprevention pill	5
Having mammography screening	4
Having a colonoscopy	3
Taking a vitamin D supplement	3
Changing diet	2
Having genetic counseling	2
Seeing a psychiatrist	2
Having vaccinations	2
Seeing a gastroenterologist	2
Consultation with breast clinic	1
Having physical therapy	1
Having a 24-h urine test	1
Having a blood test to check A1C level	1
Having a heart stress test	1
Seeing a nutritionist	1
Seeing a social worker	1
Starting an exercise program	1
Taking a blood pressure medication	1
Taking a medication for foot infection	1
Taking alternative medicine	1
Taking a mood stabilizer	1
Taking a medication to lower cholesterol	1
Taking insulin medication	1
Using an incentive spirometer	1
Using a sleep apnea machine	1
Total	41

#### Theme 2: lack of knowledge about chemoprevention

Lack of knowledge about chemoprevention is a barrier that emerged from the clinical encounter data. One provider stated that s/he was not aware of a pill for chemoprevention. Another provider strongly discouraged his/her patient from taking a chemoprevention pill, indicating that it was not needed, thereby displaying a lack of knowledge of the benefits of chemoprevention for breast cancer risk reduction. In another clinical encounter, a provider did not discuss the benefits or harms of chemoprevention and reassures the patient that her mammograms have been normal in the past. Most providers were strong advocates of screening mammography for their high-risk patients without discussing chemoprevention as an option for breast cancer risk-reduction. The excerpts (Additional file 1: Boxes 3, 4) demonstrate providers’ lack of knowledge about chemoprevention.

#### Theme 3: limited risk communication during clinical encounters

We found that another key barrier to SDM for chemoprevention during the clinical encounter is limited risk communication about breast cancer risk. While chemoprevention was briefly discussed by several providers during clinical encounters, there was minimal communication about breast cancer risk. Also, there was no discussion of the harms and benefits of chemoprevention as a primary method for reducing breast cancer risk. Instead, most providers were more comfortable ordering mammography for their patients. In one of the clinical encounters, the patient states that she was told her breast cancer risk in five years and she questions how it is possible to know her 5-year risk; however, the provider did not explain risk and began discussing his/her plans to continue to monitor the patient through yearly mammograms. In another clinical encounter, a patient asks the provider if one can prevent cancer with a drug; however, the provider did not address this question. The following clinical encounter excerpts (Additional file 1: Box 5, 6, 7) demonstrate the providers’ missed opportunity to engage in risk communication with their high-risk patients. For example, in Additional file 1: Box 7, the conversation includes a patient that says she was told that she had a 15% risk of developing breast cancer, but this number seemed low to her. However, the provider did not explain to the patient the average lifetime risk and how her 15% risk compares to an average risk woman.

## Discussion

This qualitative study used real clinical encounter data to identify provider-related barriers to shared decision-making (SDM) for chemoprevention in the primary care setting. First, we found that there are numerous competing demands occurring during a primary care visit that often forces providers to establish priorities based on their patients’ needs. Therefore, SDM for breast cancer chemoprevention seldomly occurred between PCPs and their high-risk patients during clinical encounters. There were a multitude of discussions around other primary care health topics that were occurring in the clinical encounters, yielding little time for discussion about breast cancer risk and risk-reducing options, particularly chemoprevention. Since SDM can be time-consuming, providers could benefit from using resources such as our decision support tool BNAV to help facilitate risk communication and chemoprevention discussions in a timelier and more succinct manner. Since the average age of our participants is 73 years, future considerations should include targeting younger women at higher-risk for developing breast cancer who have fewer comorbidities and are more open to taking chemoprevention medications. These younger women with a higher risk–benefit profile are more likely to benefit from taking chemoprevention in comparison to older women with lower risk. The lack of SDM that occurred in these encounters is consistent with previous studies that have focused on cancer-related care [11, 22]. Hoffman et al. [11] found that with a nationally representative sample of adults who recently faced screening decisions, most reported that their healthcare providers failed to provide balanced information, particularly about the pros and cons of cancer screening.

Another finding of this study is that providers lacked knowledge about chemoprevention, which may have prevented them from having meaningful discussions with their patients to assess interest in taking chemoprevention. In our study, providers did not feel comfortable prescribing risk-reducing chemoprevention medications, such as tamoxifen, raloxifene, or aromatase inhibitors, to women who were at high risk for developing breast cancer. Previous studies have found that insufficient knowledge among providers about anti-estrogen therapy is a major barrier to uptake of chemoprevention [23, 24]. A study conducted in the UK found that providers felt poorly informed about prevention therapy, which discouraged patient discussion on the topic [25]. Additionally, providers were not prepared to prescribe chemoprevention to their high-risk patients. Lack of knowledge about chemoprevention options precludes PCPs from prescribing these medications with confidence [24]. It is important to note that about half of the providers in our study had not yet finished training or completed training less than 5 years ago. These providers who are younger in the profession may not have received formal education about chemoprevention and have limited experience prescribing chemoprevention to high-risk women. This finding indicates the need for formal education added to curricula in medical and nursing schools regarding the effectiveness of chemoprevention as a primary prevention method and training during residency to orient providers to prescribing chemoprevention in the primary care setting. Once providers enter clinical practice they could receive continuing education credits for completing web-based educational interventions such as the *BNAV* Tool.

There is also a need to change the culture of prescribing chemoprevention in the primary care setting. Previous studies have found that exposure to direct-to-consumer advertisements of statin drugs increased the odds of patients both being diagnosed with high cholesterol and using statins by 16–20% [26]. These pharmaceutical advertisements have increased patient demand for statins [26]. Much like how PCPs are comfortable prescribing statins for hypercholesterolemia, there is the potential for providers to routinely prescribe chemoprevention as a primary prevention of breast cancer and for this to become the standard of care in primary care settings.

Additionally, we found that providers seldomly engaged in risk communication with their patients. Several options exist for breast cancer risk-reduction [27]. However, many women may be unaware of their risk status due to a provider’s inability to assess and communicate risk during the primary care clinical encounter [28]. Previous research demonstrated that providers are uneasy addressing issues raised in complex risk scenarios, particularly in a time-constrained setting [29]. Instead, clinical encounters demonstrated that providers felt more comfortable ordering mammography screening for their female patients, as opposed to discussing or prescribing risk-reducing chemoprevention. Future directions may include incorporating risk discussions into conversations about mammography screening and presenting patients with options for prevention such as chemoprevention drugs. For example, when discussing the importance of obtaining a mammogram for early detection of breast cancer, providers can also convey probabilistic risk information, when to start and stop breast cancer screening, how frequently to have a screening mammogram, and what breast imaging studies to order, including tomosynthesis, breast ultrasound, and MRI.

## Practice implications

There is a need to implement evidenced based guidelines into clinical care to promote greater patient-provider communication about breast cancer risk management approaches, such as chemoprevention drugs. Presently, very few women receive a prescription for chemoprevention by PCPs, despite national guidelines, which recommend that clinicians offer to prescribe risk-reducing chemoprevention medications to women who are at high-risk for developing invasive breast cancer [2]. Since challenges remain in implementing national guidelines into clinical practice, one consideration is disseminating information about the current guidelines at grand rounds, department meetings, and implementing provider training sessions. There is also a need to identify providers within the primary care settings who can serve as champions for cancer prevention. Furthermore, there is a need to change the standard of care by integrating web-based decision support tools designed for providers, such as the *BNAV* intervention used in this study. *BNAV* addresses provider-related barriers to chemoprevention by educating and preparing providers to assess and manage their patient’s risk of breast cancer by providing clinicians with a personalized risk profile of their patient along with educational resources on breast cancer prevention options, such as chemoprevention. Despite providers having access to *BNAV,* we found that providers were not skilled or comfortable having SDM discussions with their patients and lacked knowledge of chemoprevention. Perhaps the earlier version of *BNAV* was not well utilized by providers because it was not integrated into the clinic workflow and instead was a free-flowing website; therefore, it wasn’t widely adopted by providers initially. Since this study has been conducted, the senior investigators of the research team have integrated *BNAV* into the ambulatory medicine dashboard of the EHR to improve access to the tool for providers. Our findings underscore the need for a more practical application of decision support tools and hands on demonstration of the tool to educate and support providers and their patients in the primary care setting to reduce barriers to SDM for chemoprevention.

### Strengths and limitations

Recordings of clinical encounters allowed us to gain insight into provider-patient communication. The study also has limitations. First, we recorded single clinical encounters and did not follow these encounters over time. In addition, our study included a small number of clinical encounters that occurred at an urban academic medical center in New York City; therefore, our findings may not be generalizable to other clinical settings.

### Conclusion

In this study, we identified provider-related barriers to SDM for chemoprevention uptake among high-risk women that have important implications for future improvements. Providers need education, decision support, and resources to conduct risk assessments, engage in risk communication and SDM with their high-risk patients, and to gain confidence in prescribing chemoprevention in the primary setting.

## Supplementary Information

**Additional file 1.** BOXES 1- 7. Excerpts from audio-recordings of clinical encounter data to demonstrate qualitative themes.

## Data Availability

All data generated or analyzed during this study are included in this published article.
