# Supplementary material for: Qualitative analysis of shared decision-making for chemoprevention in the primary care setting: provider-related barriers
Source: BMC Med Inform Decis Mak. 2022 Aug 4;22:208. doi: 10.1186/s12911-022-01954-y (PMC9354269; doi:10.1186/s12911-022-01954-y)
Supplement: Supplementary file 1 — Additional file 1. BOXES 1- 7. Excerpts from audio-recordings of clinical encounter data to demonstrate qualitative themes. [file 12911_2022_1954_MOESM1_ESM.docx]

BOXES 1- 7

Box 1: ***Competing Demands during the Clinical Encounters [Clinical Encounter #6319]:***

***Patient:*** *When they did the testing for the study, they told me I have a 1 in 7 chance of getting, what is it? 7 out of 100 women who could get breast cancer.*

***Provider:*** *Yes, yes. Now we’re going to send you to have the mammogram.*

***Patient:*** *Yes. And then they said if you want to get counseling*

***Provider:*** *You can go over there and get counseling and they can do some genetic testing, do all of that.*

***Patient:*** *Yeah.*

***Provider:*** *Yeah, I mean that’s what they see it for but believe me, you do the mammogram, everything is fine, I mean, with your history, to take more pills, you don’t have to take medication, you don’t take medication the way you’re supposed to, you know that.*

***Patient:*** *I forget-*

***Provider:*** *You’re forgetful with that. I’m not even going to consider that one, so you’re not going to prevent something when I have to treat something.*

***Patient:*** *okay*

***Provider:*** *And treatment now is blood pressure.*

***Patient:*** *okay*

***Provider:*** *I’m very concerned because I saw you a year ago, with very high blood pressure. I see you now with very high blood pressure.*

***Patient:*** *mhhm*

***Provider:*** *I saw you a year ago, obese. And I see you now, with 15 more pounds. That’s my concern.*

***Patient:*** *okay*

***Provider:*** *Again, we do the mammogram, but that’s it.*

Box 2: ***Competing Demands during the Clinical Encounters [Clinical Encounter #6319]:***

*Provider: I’ll give you something for the yeast infection and the most important thing is to go to social worker to help you with oxygen and getting the insulin.*

*Patient: Hmmm.*

*Provider: And we’ll get that chest x ray today. And I’ll prescribe the Tylenol and the Naproxen. So I sent in the Naproxen and the Tylenol. That’s great. You went to all your appointments, you’re getting things done.*

*Patient: Mhmm.*

*Provider: So now there’s like very little things to get done now. It’s really just getting your Novolog, then things will be better and under control.*

Box 3***: Lack of Knowledge about Chemoprevention [Clinical Encounter #7610]:***

***Provider:*** *Alright so that’s what we’re doing. I talked to the study coordinator already.*

***Patient:*** *Yeah, they are talking about a pill to take or something. On the first sheet.*

***Provider:*** *Okay. So this is just for…I know, this is part of the study. This is why they’re recording.*

***Patient:*** *I remember them saying something about a pill.*

***Provider:*** *I don’t see anything about--*

***Patient:*** *Taking a pill?*

***Provider:*** *I don’t know anything about this pill.*

Box 4***: Lack of Knowledge about Chemoprevention [Clinical Encounter # 9163]:***

*Patient: And Dr. That has me very nervous.*

*Provider: What?*

*Patient: That I was called to do a survey about breast cancer. I don’t know, doctor…*

*Provider: That comes from the family history that you have. Who has breast cancer in your family?*

*Provider: My mother; Therefore, it comes from people who have had breast cancer in the family*

*Patient: Yes*

*Provider: They are more likely to have breast cancer and today we know whether or not. First, they will talk to you. You do not have any change in your mammograms, your mammograms have always been completely normal, but they*

*Patient: But I’m worried*

*Provider: You do not have to worry about that*

*Patient: What a relief you give me*

*Provider: At all*

*Patient: You know you’re my doctor*

*Provider: No, no, do not have to get with that, this is a research study, this is a research study. Patient: There… I want you to explain to me*

*Provider: This is a research study. They want to know what is*

*Patient: You know that you are my father*

*Provider: They want to know how much a person like you who knows that her mother had breast cancer know about your breast cancer risk. How much you know that is a genetic disease that is spread from person to person, if you have a daughter, how much has explained to your daughter who has to get screened early, that kind of thing*

*Patient: Yes*

*Provider: That kind of things, also today not for you but maybe it can be for your daughter, they have what is called prevention.*

*Patient: Uhm*

*Provider: They are trying medicine that they think if a person is likely to have breast cancer and use this medication if have genetic factors because having genetic factors make you susceptible to that then we give these medicines that maybe prevent her to develop cancer, that’s the thing, but you don’t have to worry*

*Patient: Okay*

*Provider: Because this is your mammogram recently, you understand, completely normal and all your mammograms have been completely normal all the time.*

Box 5: ***Limited Risk Communication [Clinical Encounter #9163:]***

***Patient:*** *I was told in 5 years [breast cancer risk], but I say how in 5 years? that’s what they said to me when they interviewed me.*

***Provider:*** *Well what we do in your case and usually what I do is that I keep checking your results for the next five years. You understand? In your case I will do until 6 years. I only send patients to do a mammogram until 75 years old…*

Box 6: ***Limited Risk Communication [Clinical Encounter #15326]:***

*Provider: Now, let me check some of the other test that you had, you had a mammogram last September and that looks…that looks fine. It what we call, was normal mammogram last September, ok.*

*Provider: The recommendation for something called United States Preventive Services Task Force recommends mammogram every two years*

*Patient: They change it, now is two years?*

*Provider: I am looking back in your records. In term of recommendation, there are some groups that recommend mammogram every year, there are others that recommend every two years. How about you, do you have a preference do you rather have it every year?*

*Provider: Tell about a little more, tell me about your family history risk*

*Patient: I have a few cousins that had had breast cancer, one passed away due to that and there are (three) other cousins that had breast cancer in both my mother side and my dad side and there are nieces for both my mother and father*

*Provider: Ok, so you have one cousin that developed breast cancer and passed away*

*I am going to reach out to the breast clinic to see about what else we need to do given your cousins had breast cancer, ok.*

*Patient: We’re still going to continue annual mammogram, but I want to make sure that we are not missing something else.*

Box 7: ***Limited Risk Communication [Clinical Encounter #13042]:***

*Patient: And in front of me she did graph that showed that I do, that I could have cancer. I had in my mind that people after 70 years gets cancer, there is a 15% chance for me, a 15%.*

*Provider: what do you think of that?*

*Patient: I do not think much about negative things*

*Provider: then you interpret the information as something negative*

*Patient: very negative because it can happen, but I say that to me it will not happen. They were going to try a new drug, a new drug, if I would like to try a new drug to prevent. Based on the genetic study of my family, I have 15% as she says*

*Provider: yes*

*Patient: that if I wanted to take a drug for prevention and I said no*

*Provider: And you understand the risk of 15% in your life, for you is not as high?*

*Patient: no, not for me, I think*

*Provider: Ok*

*Patient: now if they say 70% or 80% chance I think that’s it*

*Provider: ah, ok*

*Patient: 15% no, I think, I don’t know*

*Provider: So you told them that taking a new medication the risk of that*

*Patient: also, uhm*

*Provider: so you think there's risk with medicine*

*Patient: I don’t know if there’s a risk, but I told her that I was not going to take it, I told her no, to prevent cancer, no, no*

*Provider: Ok, maybe if you ...*

*Patient: can you prevent cancer with medication, can you?*

*Provider: well, that’s what the study they are doing says; that they have pill to lower the risk.*
